# Long-Term Biodistribution of Fe_3_O_4_@Au Core–Satellite Nanoparticles Assessed by CT and MRI In Vivo

**DOI:** 10.3390/ijms27146147

**Published:** 2026-07-09

**Authors:** Kristina Shpakova, Vsevolod Skribitsky, Yulia Finogenova, Anton Kasianov, Alexey Lipengolts, Angelina Skribitskaya, Anna Smirnova, Artem Laktionov, Anton Popov, Andrey Kozlov, Sergey Klimentov, Elena Grigorieva

**Affiliations:** 1 N.N. Blokhin National Medical Research Center of Oncology, Moscow 115522, Russia; shpakova.k.e@gmail.com (K.S.); skvseva@yandex.ru (V.S.); a_kasianov@mail.ru (A.K.); smirn-ova@mail.ru (A.S.); grig-elen11@mail.ru (E.G.); 2 Kurnakov Institute of General and Inorganic Chemistry, Russian Academy of Sciences, Moscow 119991, Russia; 3 Institute for Physics and Engineering in Biomedicine, National Research Nuclear University “MEPhI”, Moscow 115409, Russia; sav1998@list.ru (A.S.); aalaktionov@mephi.ru (A.L.); aapopov1@mephi.ru (A.P.); kozavr00@mail.ru (A.K.); kliment-61@mail.ru (S.K.); 4 The Loginov Moscow Clinical Scientific Center, Moscow 111123, Russia

**Keywords:** laser ablation, bioimaging, contrast agents, biodegradation, polyethylene glycol, toxicity, safety assessment, theranostics, Fenton reaction, protein corona

## Abstract

Nanoparticles combining gold and iron oxide are promising for a wide range of biomedical applications, including photothermal therapy, radiotherapy enhancement, drug delivery, and diagnostic imaging. However, their long-term biodistribution and safety profile remain largely unexplored. Here, we synthesized Fe_3_O_4_@AuNP core–satellite nanoparticles (Fe_3_O_4_@AuNPs) using femtosecond laser ablation, functionalized them with 15 kDa polyethylene glycol (PEG), and characterized them using a panel of physicochemical techniques. Healthy C57BL/6 mice received an intravenous injection of Fe_3_O_4_@AuNPs at 730 mg Au/kg and 82 mg Fe/kg, respectively. Biodistribution was monitored by computed tomography (CT) and magnetic resonance imaging (MRI) over 12 months, after which gold and iron concentrations were measured ex vivo, and long-term toxicity was assessed via histology and blood biochemistry. Nanoparticles accumulated predominantly in the liver and spleen. Quantitative analysis of CT images revealed a gradual decrease in gold content, while MRI showed a progressive reduction in negative contrast in the liver between 6 and 12 months, suggesting possible changes in the Fe_3_O_4_ core structure. Over the one-year observation period, no differences in behavior or body weight gain were detected between the treated and control groups. Histological examination revealed no pathological changes other than mild age-related alterations. These findings provide a baseline for the long-term behavior of laser-ablated core–satellite Fe_3_O_4_@AuNPs, which is essential for their further development in diagnostic and theranostic applications.

## 1. Introduction

Hybrid nanoparticles combining gold and iron oxide components (Fe_3_O_4_@AuNPs) have attracted attention due to their unique combination of functional properties. In particular, gold provides high X-ray attenuation and plasmonic effects, while Fe_3_O_4_ serves as a negative contrast agent in magnetic resonance imaging (MRI) and also enables magnetic targeting. Taking advantage of these features, Fe_3_O_4_@AuNPs can be utilized for the diagnosis and therapy of various pathological conditions [1]. For example, under near-infrared irradiation, Fe_3_O_4_@AuNPs provide effective photothermal therapy (PTT) for tumors [2]. When exposed to an external X-ray beam, gold enhances the local radiation dose, making Fe_3_O_4_@AuNPs promising radiosensitizers for radiotherapy [3]. Fe_3_O_4_@AuNPs can also be employed as a drug delivery platform, relying on active or passive targeting [4]. Moreover, several modalities can be used simultaneously, thus enabling combined therapy [5,6].

In diagnostic applications, Fe_3_O_4_@AuNPs act as contrast agents for both computed tomography (CT) and MRI [7]. Accumulating in the reticuloendothelial system (RES) via macrophage uptake, they allow the assessment of diffuse and focal lesions of the liver and spleen. Another important area is angiography for X-ray endovascular surgery, where the long circulation time of PEG-modified Fe_3_O_4_@AuNPs in the bloodstream enables vessel visualization without repeated injections of contrast agent. Currently, bimodal Fe_3_O_4_@AuNPs are primarily considered for the imaging of neoplasms: CT provides high spatial resolution and quantitative evaluation of contrast enhancement, while MRI offers excellent soft tissue imaging, which is critically important for assessing tumor extent and its relationship with adjacent structures [8]. Thus, Fe_3_O_4_@AuNPs represent a multifunctional platform that warrants further investigation in various areas of biomedicine.

Numerous studies have synthesized bimodal nanoparticles of various designs, including heterodimers of different shapes [9,10], polymeric hybridosomes [11], nanoclusters [12], nanostars [13,14] and nanoflowers [15]. However, the most frequently used configuration in biomedical research is the core–shell structure, which consists of an Fe_3_O_4_ core surrounded by a complete gold layer [16]. Gold is chemically inert and is not subject to degradation under physiological conditions. Although the Fe_3_O_4_ core is potentially biodegradable, within a core–shell structure, it is enclosed in a thick gold layer and is thus inaccessible to lysosomal enzymes. Consequently, such particles remain stable in the body for long periods.

Typically, core–shell Fe_3_O_4_@AuNPs are prepared using chemical methods. For example, a two-step synthesis can be employed: first, iron salts are precipitated to obtain Fe_3_O_4_ nanoparticles, and then gold is reduced from HAuCl_4_ onto their surface to form a complete gold shell [17]. Although highly reproducible, chemical methods require the use of surfactants and stabilizers, which may remain on the particle surface and potentially affect its biological performance.

In this study, we investigated particles of a different design, namely core–satellite particles. Unlike core–shell structures, in core–satellite particles, gold does not form a complete shell but estavlish individual satellites decorating the surface of the Fe_3_O_4_ core. As a result, the Fe_3_O_4_ surface remains partially exposed and accessible to enzymes. Therefore, core–satellite Fe_3_O_4_@AuNPs may offer the possibility of core biodegradation. Moreover, the satellites are considerably smaller than the whole bimodal particle, so after disintegration of the nanostructure, they could be partially eliminated via feces or urine.

To prepare core–satellite Fe_3_O_4_@AuNPs, we used a physical method of pulsed laser ablation in liquids (PLAL) [18]. The synthesis is carried out in two steps: first, a gold target is ablated with a femtosecond laser in an aqueous NaCl solution to produce a colloidal solution of gold nanoparticles. Subsequently, an iron target is ablated in the colloidal solution of pre-formed gold nanoparticles at reduced pulse energy, resulting in the formation of gold satellites on the surface of iron oxide particles. The main advantage of laser ablation is the absence of surfactants and other chemical contaminants—the as-prepared Fe_3_O_4_@AuNPs have an "absolutely bare" surface. This is critically important for subsequent controlled functionalization and eliminates the potential toxicity associated with residual reagents. Therefore, in this study, laser ablation was used to obtain the core–satellite Fe_3_O_4_@AuNPs.

To ensure colloidal stability under physiological conditions and prolong blood circulation time, the nanoparticle surface needs to be appropriately modified. Upon entering the bloodstream, Fe_3_O_4_@AuNPs acquire a protein corona, which leads to rapid uptake by macrophages and accumulation in the RES organs (liver and spleen) [19,20]. The most common strategy to reduce protein adsorption is coating with polyethylene glycol (PEG). PEG creates a steric barrier, thereby decreasing non-specific interactions with plasma proteins and phagocytic cells.

Here, we used PEG with an average molecular weight of 15 kDa, attached to the gold satellites via a lipoic acid linker. Lipoic acid contains two thiol groups, forming stable Au–S bonds with the gold surface. The choice of the long 15 kDa PEG chain, substantially longer than the commonly used 2–5 kDa chain, was dictated by the relatively large size of Fe_3_O_4_@AuNPs (diameter of approximately 90 nm). According to Perrault et al. [21], larger nanoparticles require longer PEG chains to maintain prolonged blood circulation. Prolonged circulation makes accumulation in the liver more gradual and enables dynamic monitoring of the biodistribution process over time. We used two bioimaging modalities for the non-invasive study of nanoparticle behavior in vivo: CT allowed for quantitative determination of gold concentration in tissues, while MRI was used as an indirect approach to evaluate possible changes in the magnetic Fe_3_O_4_ core.

The high gold dose used here (730 mg/kg) was chosen for two reasons. On the one hand, CT imaging and radiosensitization both demand gold doses of at least 400–500 mg/kg [22,23,24,25]. On the other hand, the one-year design of our study required a sufficiently high initial nanoparticle load to ensure that gold and iron signals remained detectable after months of possible clearance or core degradation. However, such a high administered dose inevitably raises the concern of long-term toxicity. Therefore, we performed a one-year observation in healthy mice and conducted endpoint histological and biochemical analyses to evaluate the safety profile of Fe_3_O_4_@AuNPs.

The aim of this study was to synthesize and characterize Fe_3_O_4_@AuNPs with a core–satellite structure stabilized with PEG (15 kDa), to investigate their biodistribution via CT and MRI, and to assess the tolerability in healthy mice at a dose that enabled long-term bimodal monitoring.

## 2. Results

### 2.1. Characterization of Fe_3_O_4_@AuNPs and Phantom Studies

**Figure 1:**
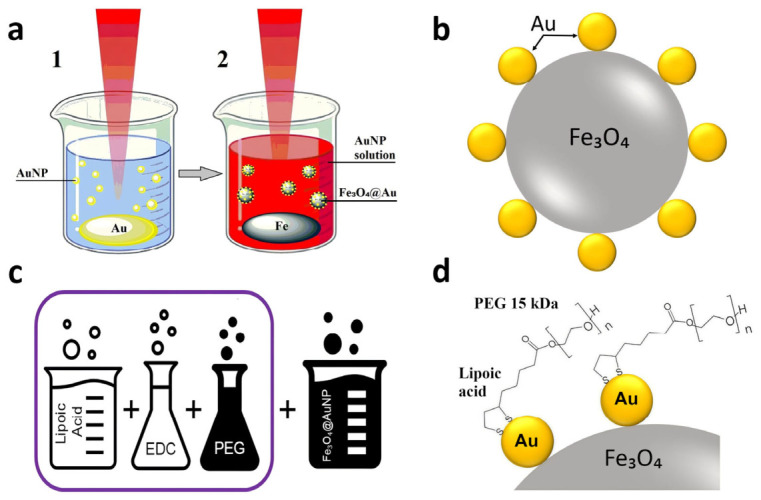
Schematic illustration of Fe_3_O_4_@AuNPs synthesis and functionalization. (**a**) Two-step laser ablation synthesis: first, generation of small Au nanoparticles; second, ablation of an Fe target in the presence of pre-formed Au colloid to form Fe_3_O_4_@Au core–satellite nanoparticles; (**b**) schematic representation of a single core–satellite nanoparticle showing an Fe_3_O_4_ core decorated with Au satellites; (**c**) synthesis of the LA–PEG conjugate via carbodiimide chemistry. (**d**) Surface functionalization: lipoic acid provides two thiol groups that form stable Au–S bonds with the gold satellites, anchoring 15 kDa PEG chains.

The synthesis of Fe_3_O_4_@Au core–satellite nanoparticles and their subsequent functionalization with LA–PEG are illustrated in Figure 1. A two-step laser ablation approach was used to prepare the core–satellite structure (Figure 1a): first, gold nanoparticles were produced by femtosecond laser ablation of a gold target, followed by ablation of an iron target in the presence of the pre-formed gold colloid. The resulting nanoparticles consisted of an iron oxide core decorated with small gold satellites (Figure 1b). After magnetic separation to isolate the Fe_3_O_4_@AuNPs from the unbound gold, the nanoparticles were functionalized with a 15 kDa polyethylene glycol (PEG) chain using lipoic acid (LA) as a linker. The LA–PEG conjugate was synthesized in advance via carbodiimide chemistry, as illustrated in Figure 1c. The lipoic acid moiety provides two thiol groups, forming stable Au–S bonds with the gold surface, thereby anchoring the PEG chains to the satellites (Figure 1d). Detailed synthesis and functionalization protocols are provided in the Section 4.

**Figure 2:**
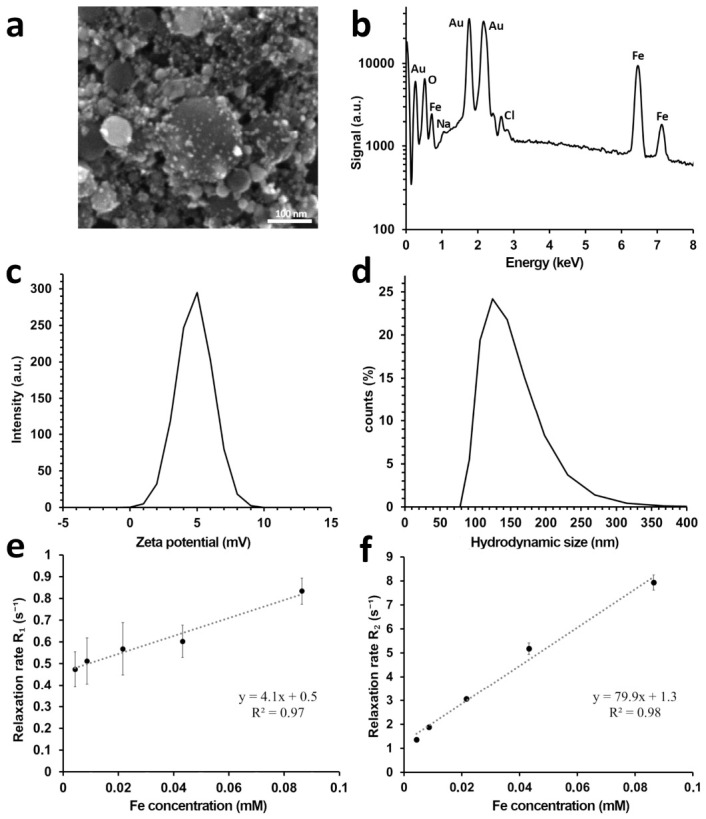
Physicochemical characterization of Fe_3_O_4_@AuNPs: (**a**) representative SEM image showing the core–satellite morphology (scale bar = 100 nm); (**b**) EDX spectrum confirming the presence of Fe, Au, O, Na, and Cl; (**c**) zeta-potential distribution in PBS (pH 7.4) showing a near-neutral charge (4.9 ± 3.3 mV); (**d**) hydrodynamic diameter distribution by DLS (mean 144 ± 80 nm); (**e**) longitudinal relaxivity (r_1_ = 4.1 ± 0.7 (mM·s)^−1^); (**f**) transverse relaxivity (r_2_ = 79.9 ± 3.9 (mM·s)^−1^).

The morphology and structure of the synthesized nanoparticles were examined via scanning electron microscopy (SEM). The images revealed a core–satellite architecture, with a central iron oxide core (mean diameter, 73 ± 20 nm) surrounded by multiple smaller gold satellites (mean diameter, 8 ± 2 nm) (Figure 2a). Additionally, Figure S1 presents SEM images of Fe_3_O_4_@AuNPs before and after PEG functionalization, clearly showing the acquisition of the polymer coating.

The optical properties of the nanoparticles were assessed by UV–Vis spectrophotometry. The spectrum exhibited a surface plasmon resonance peak at 540 nm, typical of gold nanoparticles (Figure S2). Energy-dispersive X-ray spectroscopy (EDX) confirmed the elemental composition of the nanoparticles before functionalization, showing characteristic signals for iron (from the core) and gold (from the satellites), along with oxygen, sodium, and chlorine (Figure 2b). The sodium and chlorine signals originate from the NaCl solution used during laser ablation.

Fourier-transform infrared (FTIR) spectroscopy confirmed the successful attachment of LA–PEG. The spectrum showed characteristic bands of polyethylene glycol and an amide linkage, together with a C–S band indicative of thiolate binding to the gold surface, while the absence of S–S and S–H bands confirmed complete chemisorption of the lipoic acid anchor (Figure S3). The zeta potential in phosphate-buffered saline (PBS, pH 7.4) was 4.9 ± 3.3 mV (Figure 2c). The near-neutral zeta potential is consistent with a PEG-coated surface, suggesting the potential for prolonged blood circulation. Dynamic light scattering (DLS) measurements yielded a hydrodynamic diameter of 144 ± 80 nm (PDI = 0.184) (Figure 2d), which is substantially larger than the core diameter seen by SEM, as expected for a 15 kDa PEG coating. Despite the relatively wide distribution of hydrodynamic size, a PDI < 0.2 indicated a monodisperse system [26]. The concentrations of gold and iron in the final colloidal solution were determined by an ICP-OES level of 74.5 ± 1.2 mg/mL for gold and 8.4 ± 0.2 mg/mL for iron.

The relaxivity properties of Fe_3_O_4_@AuNPs were evaluated by MRI at 3 T. The longitudinal relaxivity (r_1_) was 4.1 ± 0.7 (mM·s)^−1^ (Figure 2e), and the transverse relaxivity (r_2_) was 79.9 ± 3.9 (mM·s)^−1^ (Figure 2f). The high r_2_/r_1_ ratio (~19.5) indicates that the nanoparticles behave as a negative contrast agent, consistent with response of the iron oxide core.

To enable quantitative CT imaging, a calibration curve was established using a phantom containing Fe_3_O_4_@AuNP solutions of known gold concentrations. The increase in radiodensity (ΔHU) relative to water was plotted against the gold concentration, and the data were fitted with a linear function (Figure S4). The resulting calibration equation was

[Au] = 0.0121 × ΔHU,(1)

where [Au] is the gold concentration in mg/mL, and ΔHU is the radiodensity increase. Detailed phantom preparation and scanning parameters are described in the Methods Section 4.4.

### 2.2. Acute Toxicity

The safety and biodistribution of Fe_3_O_4_@AuNPs were examined in healthy female C57BL/6 mice following a single intravenous injection at doses of 730 mg Au/kg and 82 mg Fe/kg, respectively. During the initial hours after administration, none of the treated animals displayed any indications of acute toxicity, such as abnormal behavior, pain-related signs, unconsciousness, or death.

Visible areas of the body, including the paws and tail, showed slight skin darkening (Figure S5). Nevertheless, this effect was much weaker than that documented for pure gold nanoparticles at similar gold loads [27]. Animal welfare and general behavior remained unaffected despite the skin discoloration. To evaluate possible short-term physiological consequences, body weight was recorded for both the experimental and control groups over 21 days, with similar growth trends observed between the two groups (Figure S6). Collectively, these observations demonstrate that the administration of Fe_3_O_4_@AuNPs was not associated with overt signs of acute toxicity, thus enabling further in vivo investigations.

### 2.3. Short-Term Biodistribution

Owing to the bimodal contrast properties of Fe_3_O_4_@AuNPs, their in vivo behavior could be monitored non-invasively by CT and MRI. However, MRI does not allow quantitative assessment of contrast agent concentration. Therefore, biodistribution was evaluated by quantifying gold concentrations from CT data, as tissue radiodensity in CT images is directly proportional to the gold content. Since CT quantifies the signal per voxel volume, gold concentrations were expressed per unit volume of tissue (mg/cm^3^).

**Figure 3:**
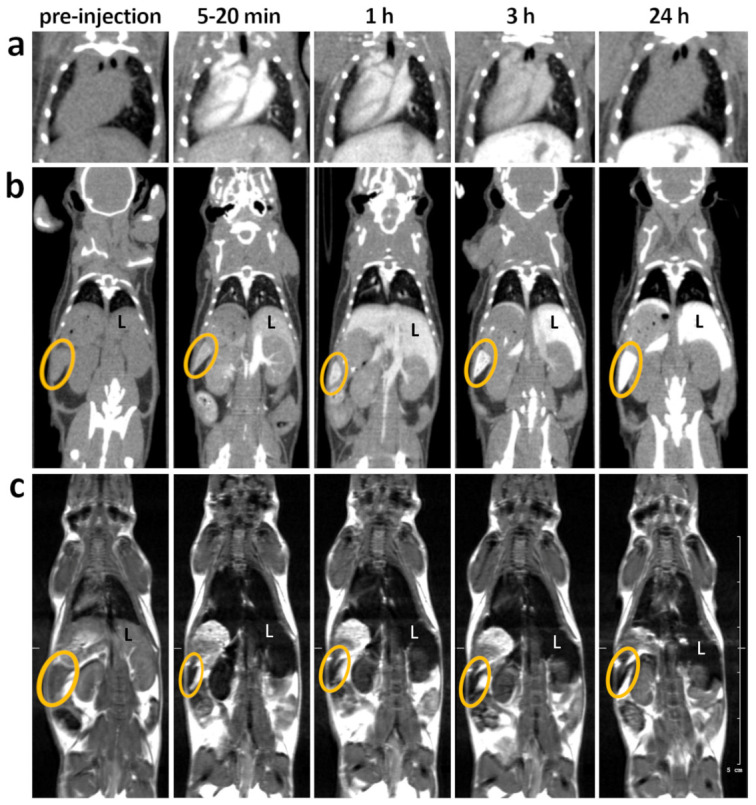
CT and MRI images of mouse organs (coronal views) before injection and at several time points up to 24 h after Fe_3_O_4_@AuNP administration. (**a**) CT images of the heart; (**b**) CT images of the liver, spleen, and kidneys; (**c**) MRI images of the liver, spleen, and kidneys. The orange ring indicates the spleen; the letter L indicates the liver.

Following intravenous administration, the nanoparticles are located in the mouse blood. Therefore, on CT images of the heart, the chambers appear contrast-enhanced, and the interventricular septum is clearly visible against this background (Figure 3a). Cardiac chamber enhancement was the highest immediately after injection, decreased substantially by 3 h, and was no longer detectable by 24 h. On MRI, the heart signal became strongly negative after nanoparticle administration, and the heart could not be visualized against the dark lung fields.

Immediately after injection, MRI showed a sharp signal decrease in the kidneys, which returned to near baseline by 24 h (Figure 3c). The rapid return of the kidney signal to baseline values suggests that the nanoparticles do not specifically accumulate in the renal parenchyma. On CT, no obvious contrast enhancement was observed in the kidneys, except for in the vessels, which were brightly delineated due to the presence of gold in the blood (Figure 3b). Furthermore, no signal changes were detected in the renal pelvises in either modality, leading to the conclusion that Fe_3_O_4_@AuNPs are not excreted with urine. This is further supported by the absence of contrast in the urinary bladder on both CT and MRI images (Figure S7). Taken together, these findings indicate no evidence of renal excretion for the studied Fe_3_O_4_@AuNPs, which is consistent with their relatively large size, substantially exceeding the pore size of the renal filtration barrier.

The most pronounced contrast enhancement in both MRI and CT was observed in the liver and spleen, which aligns with the established concept that nanoparticles accumulate in the RES organs via macrophage uptake. On MRI, the signals from the liver and spleen became strongly hypointense within the first minutes after injection (Figure 3c). Unlike MRI, CT provides a signal that remains proportional to gold concentration over a wide range, which allowed a more detailed investigation of the accumulation dynamics in these organs. CT imaging revealed a gradual increase in contrast enhancement in the liver and spleen during the first 24 h post-injection (Figure 3b). Subsequently, both CT and MRI patterns remained stable from 24 h to 1 month in all studied organs (Figure S8).

**Figure 4:**
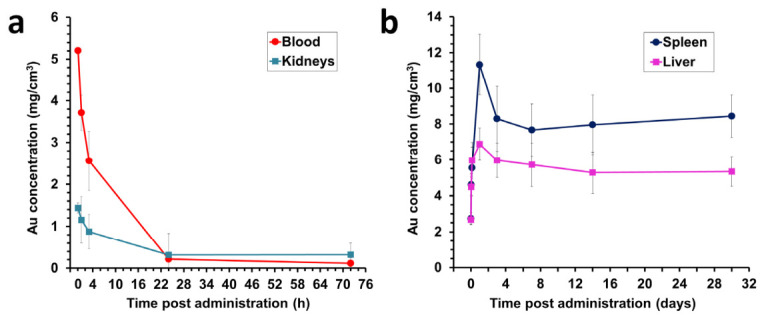
Short-term biodistribution: quantitative CT analysis. (**a**) Gold concentration in blood and kidneys (mg/cm^3^) over the first 72 h after intravenous injection of Fe_3_O_4_@AuNPs; (**b**) gold concentration in the liver and spleen (mg/cm^3^) over the first month post-injection. Data are presented as mean ± SD.

Quantitative CT analysis of blood revealed a maximum gold concentration of 5.2 ± 0.4 mg/mL immediately after injection, which then dropped to no more than 0.2 ± 0.1 mg/mL by 24 h (Figure 4a). The blood half-life of the nanoparticles was estimated to be 2.9 ± 0.2 h.

In the renal parenchyma, a moderate increase in radiodensity was detected, with gold concentrations reaching 1.4 ± 0.5 mg/cm^3^. The gold concentration then declined rapidly, falling to no more than 0.3 ± 0.2 mg/cm^3^ by 24 h, without any further changes (Figure 4a).

In the liver and spleen, the maximum gold concentration at 24 h post-injection was 6.9 ± 0.8 mg/cm^3^ and 11.3 ± 1.7 mg/cm^3^, respectively. Thereafter, the gold content in both organs decreased slightly by day 7 and then remained stable up to one month (Figure 4b).

In other organs, including the brain and muscles, no visible contrast enhancement was observed with either modality. Quantitative CT analysis likewise revealed no increase in radiodensity, indicating that gold concentrations remained below the detection limit of the method.

### 2.4. Long-Term Biodistribution

**Figure 5:**
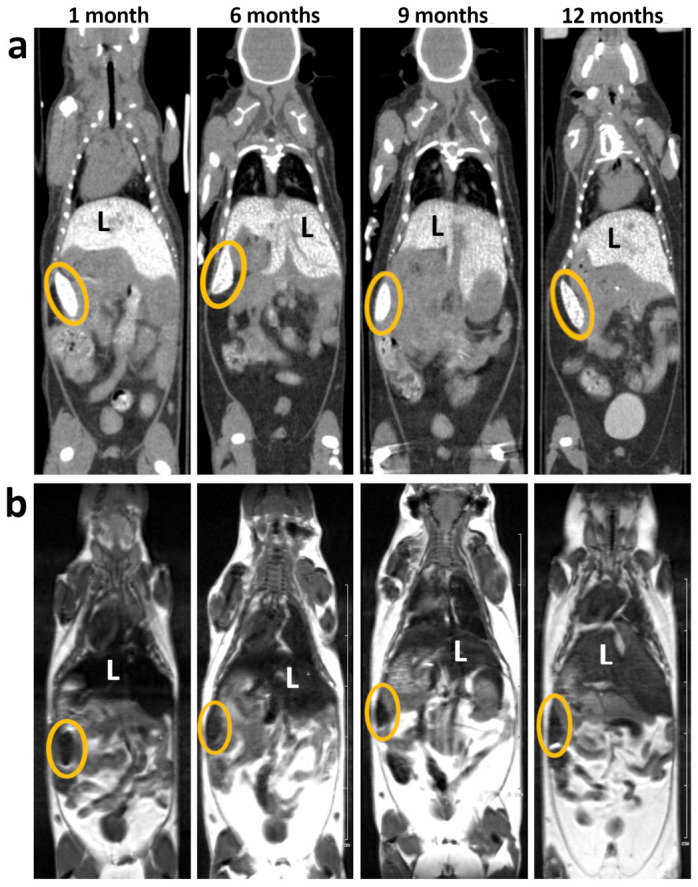
CT and MRI images of mouse organs (coronal views) at 1, 6, 9, and 12 months after intravenous injection of Fe_3_O_4_@AuNPs. (**a**) CT images of the liver and spleen; (**b**) MRI images of the same organs at the same time points. A progressive reduction in negative contrast is observed in the liver between 6 and 12 months, suggesting possible alterations in the Fe_3_O_4_ core. The orange ring indicates the spleen; the letter L indicates the liver.

**Figure 6:**
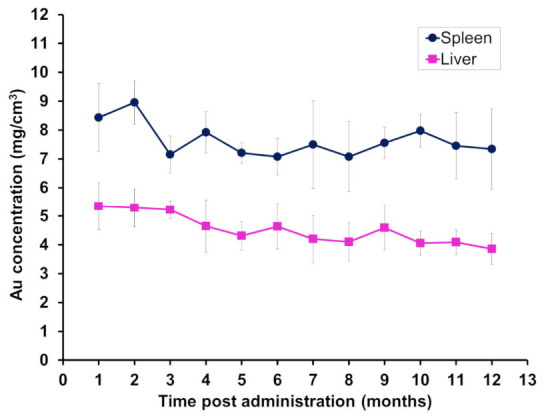
Long-term biodistribution: quantitative CT analysis. Gold concentration in the liver and spleen (mg/cm^3^) over 12 months after intravenous injection of Fe_3_O_4_@AuNPs. Data are presented as mean ± SD.

To evaluate the long-term biodistribution of the studied Fe_3_O_4_@AuNPs, the animals were monitored for one year, with CT and MRI scans performed monthly (the full set of representative monthly images is shown in Figure S9). Over the 12-month period, no major changes in biodistribution were observed compared with the results at the one-month time point: the nanoparticles produced strong contrast enhancement in the liver and spleen, with no detectable signal in other organs. However, the pattern of contrast enhancement in the liver changed over time. CT imaging revealed a gradual decrease in liver radiodensity, which was most pronounced between 1 and 4 months. Concurrently, the initially homogeneous enhancement became increasingly granular (Figure 5a). Quantitative CT analysis confirmed that gold was retained in the liver throughout the year, with a slightly declining trend in concentration (Figure 6).

On MRI, the liver signal remained hypointense relative to the native scan throughout the observation period. However, the degree of negative contrast decreased substantially between 6 and 12 months. Although the MRI signal cannot be quantified, these changes are compatible with a gradual reduction of magnetic material in the liver, which may reflect Fe_3_O_4_ core degradation and possible elimination over time (Figure 5b).

In the spleen, signal changes were less pronounced than those in the liver: MRI showed no visible differences throughout the year (Figure 5b). On CT, both visual assessment and quantitative analysis revealed a slight decrease in radiodensity during the first 1–3 months, after which no further changes were observed up to 12 months (Figure 5a and Figure 6).

After the final scan, all Fe_3_O_4_@AuNP-treated and control animals were euthanized, and organ samples were collected to determine iron and gold concentrations by ICP-OES. Gold is not naturally present in the body; accordingly, no gold was detected in any control samples, but it was detected in all organs and tissues of Fe_3_O_4_@AuNP-treated animals. As expected, the highest concentrations were found in the liver and spleen. Low gold levels were detected in the kidneys, lungs, skin, and muscles, consistent with the absence of specific accumulation mechanisms in these tissues.

In contrast, iron compounds play essential physiological roles, and iron was present in all samples from both the control and treated mice. In the spleens, livers, kidneys, lungs, and brains of treated animals, iron concentrations were significantly higher than in the controls (*p* < 0.05), reflecting the presence of the administered Fe_3_O_4_@AuNP (Table 1). No differences were observed in muscle tissue, and in the skin, the iron concentration was slightly lower in the treated group—a finding that is unlikely to be biologically meaningful and may reflect inter-individual variability rather than a treatment-related effect.

Notably, ICP-OES allowed quantification of gold concentrations in the kidneys and brain, as these levels were below the detection limit of CT, and also enabled the analysis of organs that cannot be assessed by CT. For example, the skin cannot be reliably delineated on CT images, and the lungs cannot be evaluated due to their inherently low radiodensity. However, as demonstrated by the comparison of ICP-OES and CT data for the liver and spleen, in parenchymal organs with high gold accumulation, CT allows sufficiently accurate concentration measurement. Overall, CT exhibits lower sensitivity compared with that of ICP-OES or ICP-MS, but its main advantage lies in the ability to repeatedly scan the same animal over time, enabling longitudinal individual monitoring.

### 2.5. Long-Term Toxicity

**Figure 7:**
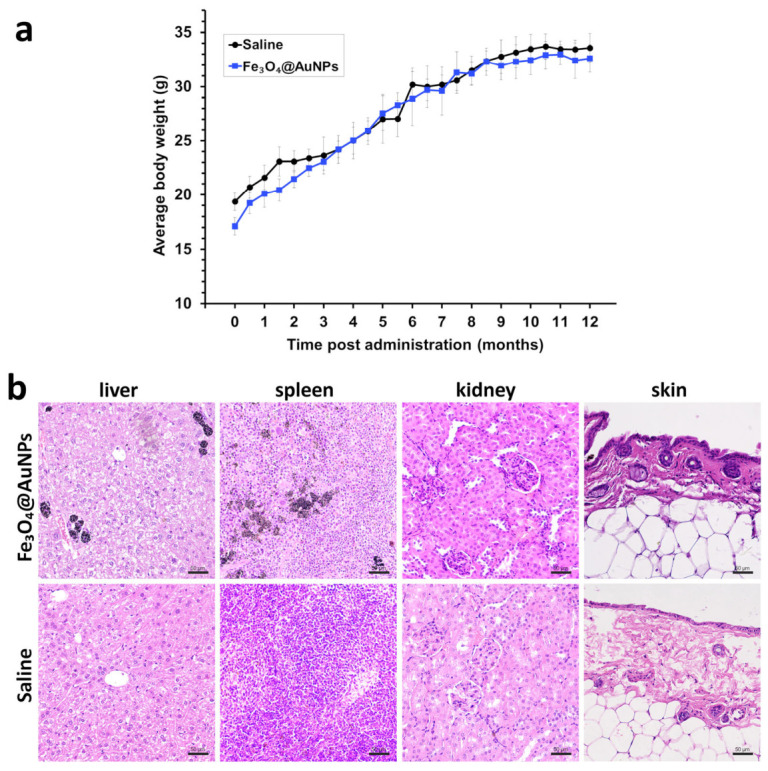
Long-term toxicity assessment. (**a**) Body weight dynamics of Fe_3_O_4_@AuNP-treated and control mice over the 12-month observation period. Data are presented as mean ± SD. (**b**) Representative histological sections of the liver, spleen, kidney, and skin from Fe_3_O_4_@AuNP-treated and control mice. H&E staining. Scale bar = 50 µm.

All mice in both the Fe_3_O_4_@AuNP-treated and control groups survived the 12-month observation period. The visible skin of the treated animals (such as the paws and tail) remained slightly darker than that of controls, with no progression over time. No differences between the groups were noted in regards to water or food consumption, coat condition, or general behavior. Body weight was recorded biweekly for all animals. As illustrated in Figure 7a, the dynamics of body weight gain were similar for the treated and control groups over the one-year period.

To assess possible adverse effects related to the prolonged retention of Fe_3_O_4_@AuNPs in the body, the mice were euthanized after the final CT scan, one year after intravenous administration. Blood was collected for biochemical analysis, and tissue samples were taken for histological examination.

Histology provided information on nanoparticle distribution and tissue morphology (Figure 7b). In the liver, both groups exhibited similar features consistent with age-related changes. Moderate vacuolization of the hepatocyte cytoplasm, indicative of age-associated lipid infiltration (steatosis), was observed in both the control and treated animals, which was not related to nanoparticle administration. In the treated group, abundant Fe_3_O_4_@AuNPs deposition was observed, predominantly located within the sinusoidal lumina as discrete clusters and inside the Kupffer cells as dense agglomerates. Importantly, the architecture of the hepatic cords remained intact. No signs of necrosis, inflammatory infiltration, cholestasis, or fibrosis were detected.

In the spleen, Fe_3_O_4_@AuNPs selectively accumulated in the red pulp, reflecting macrophage phagocytosis, while the white pulp remained unaffected. This accumulation was not accompanied by any evidence of toxic damage, such as necrosis, fibrosis, inflammation, or atrophy of lymphoid follicles. Thus, the observed pattern corresponds to the expected uptake by RES organs via phagocytosis. In the control group, occasional macrophages containing hemosiderin were noted in the red pulp, a typical finding in aged mice. Since these changes were observed only in the control group, they cannot be attributed to biodegradation of the nanoparticle Fe_3_O_4_ core.

In the skin of treated mice, sparse nanoparticle clusters were occasionally observed in the dermal interstitium, explaining the slight skin discoloration. No tissue reaction was associated with their presence. In the kidneys, lungs, and muscle tissue, no nanoparticle accumulation nor pathological alterations were found (Figure 7b and Figure S10). The tissue architecture remained intact in both groups.

Overall, nanoparticle accumulation was confined to the RES organs. Age-related changes were noted in the liver and spleen of both groups. In summary, administration of the nanoparticles did not induce any specific histological alterations in the treated animals when compared with the results for the controls.

To further assess organ function, blood biochemical analysis was performed. For most parameters, no statistically significant differences were detected between the experimental and control groups (Table 2). In particular, ALT, AST, total bilirubin, and direct bilirubin levels did not differ between the groups (*p* > 0.05). These findings suggest that liver function was not markedly affected.

The only parameter that showed a statistically significant difference between groups was creatinine (*p* = 0.035). Although higher levels were observed in the experimental group, the absolute elevation was modest. Notably, creatinine levels in the control group also exceeded the reference range for healthy mice (5–67 µmol/L) [28]. Urea levels were also slightly elevated compared to the reference range (3.2–9.3 mmol/L) but did not differ between the experimental and control groups. As previously described, histology revealed no nanoparticle accumulation or pathological alterations in the renal tissue. Taken together, these findings suggest that the elevated creatinine level is most likely attributable to age-related changes.

## 3. Discussion

In this study, we performed a long-term investigation of the biodistribution and safety profile of core–satellite Fe_3_O_4_@AuNPs following high-dose administration in healthy mice. The dose was selected based on the manufacturer’s recommendation for AuroVist™ (Nanoprobes), a gold nanoparticle CT contrast agent for small animals (~1000 mg/kg) [29], and was slightly reduced to 730 mg/kg, based on our previous experience [30]. Since gold and iron are intrinsically linked in our core–satellite nanoparticles, the high gold dose inevitably resulted in a high iron dose (82 mg/kg). For MRI-only applications, a much lower iron dose (2–10 mg/kg Fe) would have been sufficient [31,32]. However, this iron load is not a limitation of the study design but a consequence of the bimodal nature of the particles. This study is exploratory and focuses on the long-term dynamics of biodistribution; therefore, the high dose was necessary to ensure reliable CT contrast over 12 months and enable quantitative longitudinal monitoring. Each specific biomedical application of Fe_3_O_4_@AuNPs—whether as a contrast agent, radiosensitizer, photothermal agent, or drug delivery platform—requires its own studies, with doses adjusted according to the particular research aim. In such cases, CT imaging may be omitted entirely if it is not required by the study objectives [33,34,35].

Several studies performed in vivo CT and MRI following the intravenous administration of Fe_3_O_4_- and Au-containing nanoparticles. Despite significant MRI contrast enhancement in the organs, CT images showed poor contrast. For example, Griaznova et al. used laser-ablated core–satellite Fe_3_O_4_@AuNPs in BALB/c mice bearing subcutaneous EMT6 tumors at a dose of approximately 16 mg Au/kg. After intravenous administration, no contrast enhancement of internal organs was observed; tumor visualization was achieved only after intratumoral injection [36]. Li et al. investigated the contrast properties of composite Fe_3_O_4_@AuNPs. Although the authors claimed dual-mode imaging, CT and MRI were performed on different animals: MRI on C57 mice and CT on Sprague-Dawley rats. To achieve CT enhancement, rats were injected with 49 mg Au/kg, which resulted in a liver radiodensity increase of only 26 ΔHU [37]. Similarly, Caro et al. synthesized spiky core–shell Fe_3_O_4_@AuNPs and injected them into healthy Wistar rats at a dose of 19 mg Au/kg. The maximum increase in radiodensity, achieved 1 h post-injection, was only 28 ΔHU, which is insufficient for reliable diagnostic imaging [38].

Significant contrast enhancement in both modalities was achieved by Nguyen et al. using core–shell Fe_3_O_4_@AuNPs [39]. The same solution ([Fe] = 8.33 mg/mL, [Au] = 2.85 mg/mL) was used for both CT and MRI; however, the injected volume for CT was three times higher, resulting in a gold dose of approximately 39 mg/kg. The radiodensity increase in the liver was 64 ΔHU. Convincing results were also obtained by Sun et al., who employed a distinct nanoformulation consisting of 100 nm micelles loaded with gold and iron oxide nanoparticles. The preparation was administered to mice at a dose of up to 300 mg Au/kg, leading to a strong visible enhancement of the heart, blood vessels, and liver. However, no quantitative evaluation was performed [40].

In this study, we observed a much more pronounced increase in radiodensity: the maximum contrast enhancement in the liver at 24 h post-injection reached 569 ΔHU, and in the spleen, 936 ΔHU. Although this level of enhancement exceeds the diagnostic requirements, our primary goal was not only to demonstrate the contrast properties, but also to investigate long-term biodistribution. Accordingly, CT and MRI were used not as diagnostic modalities in the conventional sense but as methods for studying nanoparticle biodistribution. The high administered dose of Fe_3_O_4_@AuNPs enabled non-invasive monitoring of both the iron oxide and gold components over one year post-administration. To our knowledge, the longest observation period in the literature, reported by Caro et al., lasted only 7 days [38].

Throughout the long-term study, a gradual decrease in MRI contrast enhancement was observed in the liver. Meanwhile, on CT images, liver radiodensity remained visually stable over the one-year period, although a granular pattern emerged from 3 months onwards. This is consistent with progressive aggregation of gold satellites within the Kupffer cells, as supported by histological findings and previous reports on nanoparticle clustering in the liver [41]. Importantly, the MRI signal changes occurred later (from 6 months onwards) and remained homogeneous, with no granular appearance. Since the CT and MRI changes differed in their timing and visual pattern, they are unlikely to reflect the same underlying process. Thus, the simple aggregation or redistribution of intact Fe_3_O_4_@AuNPs cannot fully explain these observations. The aggregation of iron oxide nanoparticles would be expected to either preserve or enhance local hypointensity, not to produce the signal recovery observed in our study. Redistribution of intact nanoparticles from the liver to other tissues is unlikely given their size (>60 nm) and the established RES retention mechanism; moreover, such redistribution would have resulted in a marked decrease in CT radiodensity as well, which was not observed. The intraorgan redistribution between hepatocytes would not have altered the MRI signal because of the rather low spatial resolution of this modality (voxel size 0.2 × 0.2 × 1.0 mm).

We therefore propose that the CT and MRI findings reflect distinct processes, i.e., gold aggregation (CT) and a gradual loss of magnetic material from the Fe_3_O_4_ core (MRI). We suggest a two-stage model: following intravenous administration, Fe_3_O_4_@AuNPs are phagocytosed by macrophages and sequestered in phagolysosomes, where they lose their PEG coating and rapidly aggregate. Subsequently, the iron oxide core undergoes very slow degradation, releasing iron ions that enter normal metabolic pathways, and are ultimately excreted, predominantly in feces rather than in urine [42]. The gold satellites are not degraded and continue to aggregate within the macrophages over time, accounting for the granular CT pattern. A similar model was described by Kolosnjaj-Tabi et al., who studied heterostructures (a gold core with iron oxide petals) over one year using transmission electron microscopy (TEM) of liver and spleen sections. Despite the different architecture of their particles, the key similarity is that the iron oxide surface was accessible to enzymes. This supports our interpretation that MRI signal recovery reflects the slow loss of magnetic material, while the CT granularity is consistent with long-term aggregation of gold [43].

Given the high administered dose and the extended retention of Fe_3_O_4_@AuNPs in the liver and spleen, we next evaluated their potential toxic effects. Potential toxic effects of Fe_3_O_4_@AuNPs may be related to both the iron oxide core and the gold shell. The most comprehensive data were obtained from studies of core–shell Fe_3_O_4_@AuNPs [44]. Li et al. reported that the median lethal dose (LD_50_) of core–shell Fe_3_O_4_@AuNPs following intraperitoneal administration in mice was 8390 mg of total particle mass per kg of body weight. In a subsequent experiment, core–shell Fe_3_O_4_@AuNPs were injected into beagle dogs via liver injection at a dose of 100 mg/kg. After four weeks, histological analysis revealed no pathological changes in the examined organs (heart, liver, spleen, lung, kidney, brain). Blood biochemical analysis also showed no differences from the levels for the controls (ALT, AST, urea, creatinine). Thus, a high biocompatibility of core–shell Fe_3_O_4_@AuNPs was demonstrated [45].

Our core–satellite Fe_3_O_4_@AuNPs may potentially exhibit higher toxicity than that of core–shell nanoparticles due to the exposed surface of the Fe_3_O_4_ core [46]. The surface of iron oxide can release Fe ions, which trigger the Fenton reaction [47]. This leads to the intracellular production of reactive oxygen species (ROS), causing oxidative stress. The consequences may include mitochondrial dysfunction, cell membrane damage, inflammation, or apoptosis [48]. Therefore, after one year of animal monitoring, we performed endpoint biochemical and histological analyses. Interpretation of the data was complicated by the inevitable age-related changes associated with such a long observation period, although the control group consisted of mice of the same age.

According to histological analysis, despite the high administered dose of Fe_3_O_4_@AuNPs, nanoparticle clusters were found only in the liver and spleen. Apart from age-related steatosis, no pathological changes were detected in these organs. This is likely because the nanoparticles were located almost exclusively within macrophages and therefore, they did not interact with the epithelial cells. Importantly, no signs of fibrosis were observed in any of the examined organs, indicating the absence of chronic inflammation. Acute inflammatory responses, however, are inherently transient. Iancu et al. observed inflammatory changes and focal cellular alterations in the lungs, liver, spleen, and cardiac muscle of Wistar rats following administration of core–shell Fe_3_O_4_@AuNPs; these changes resolved by day 14 [49]. Due to the design of our study, we could not detect such changes, as histological and biochemical analyses were performed only once, one year after Fe_3_O_4_@AuNP administration.

In addition to the expected accumulation in the RES organs, ICP-AES analysis of the brain revealed detectable levels of gold and slightly elevated iron levels in the treated group compared with the levels of the controls. Although the absolute values were extremely low, they were consistently above the background. The detection of gold in the brain after intravenous administration has been well documented for small gold nanoparticles. For example, Sonavane et al. reported that 15 nm and 50 nm AuNPs accumulated in the mouse brain at 9.95 and 9.12 µg/g, respectively, 24 h after a 1000 mg/kg dose [50]. Similarly, De Jong et al. detected 0.3% of the injected dose of 10 nm AuNPs in the rat brain [51]. More relevant to our study, Chen et al. demonstrated that PEGylated SPIO–Au nanoparticles (hydrodynamic diameter ~38–76 nm)—which consist of an iron oxide core, a gold shell, and PEG coating, structurally similar to our Fe_3_O_4_@AuNPs—crossed the blood–brain barrier even in the absence of magnetic stimulation, with approximately 3.7% of the gold concentration in the blood reaching the brain [52]. Our Fe_3_O_4_@AuNPs are considerably larger than those used in the above studies (hydrodynamic diameter, 144 ± 80 nm), and we did not expect significant brain accumulation. However, the gold satellites decorating the iron oxide core have a diameter of 8–10 nm and could potentially detach from the core, contributing to the trace gold levels detected. At the same time, the iron signal may reflect gradual release of ionic iron from the core over time, resulting in its subsequent delivery to the brain via the bloodstream. Together, these two processes—satellite detachment and iron ion release—offer the most plausible explanation for the observed brain metal levels. A less likely alternative is the transcytosis of intact nanoparticles across the blood–brain barrier, which would be unexpected given the large size of the particles. Ultrastructural analysis (e.g., TEM of brain tissue) would be required to distinguish between these mechanisms, but this was beyond the scope of the present study. Additionally, we did not perform a histological analysis of the brain tissue or conduct specific neurological tests, which would be required to definitively rule out neurotoxicity, in this study. Nevertheless, throughout the 12-month observation period, the treated mice showed no signs of neurological impairment, such as seizures, ataxia, or paralysis, and their activity and behavior remained normal.

We also detected minimal amounts of nanoparticles in the connective tissue of the skin, explaining the discoloration of visible areas. Importantly, this discoloration was mild, in contrast to the dark blue color previously reported for AuNPs [27,30,53]. Skin discoloration is one of the known challenges for the clinical translation of AuNP applications; thus, Fe_3_O_4_@AuNPs appear to have an advantage in this regard.

This study had several limitations. First, the conclusion regarding Fe_3_O_4_ core alterations is based solely on qualitative MRI signal changes, which are indirect evidence. We did not perform direct structural characterization of the nanoparticles in tissues (e.g., TEM of liver sections) or biochemical analysis of urine and feces to determine the chemical form of excreted iron. Therefore, while the MRI findings are consistent with a loss of magnetic material from the core, we cannot definitively confirm the stability or biodegradation of Fe_3_O_4_@AuNPs under physiological conditions. Second, the toxicity assessment was limited to standard histological examination and blood biochemistry. We did not evaluate oxidative stress markers, inflammatory cytokines, or immune responses that could arise in response to ROS generation. Since all animals were euthanized only at the one-year endpoint, we could not detect transient biochemical or histological changes that might have occurred at earlier time points. Moreover, we did not perform a histological analysis of the brain tissue or neurological tests, which would have been informative given the ICP-AES findings in the brain. Future studies should address these limitations by investigating the longitudinal stability of Fe_3_O_4_@AuNPs in vivo, as well as their short-term safety profile through dedicated toxicity studies within the first 1–14 days post-administration, including assessment of inflammatory markers, immune responses, and neurological function.

## 4. Materials and Methods

### 4.1. Synthesis of Fe_3_O_4_@Au Core–Satellite Nanoparticles

The synthesis was performed using a two-step approach based on laser ablation of an iron target in a pre-prepared colloidal solution of gold nanoparticles (AuNPs), as described elsewhere [54]. First, the AuNPs were synthesized by femtosecond laser ablation of a gold target (99.99%) in an aqueous solution of NaCl (180 μM). A Yb:KGW laser (TETA 10, Avesta, Moscow, Russia) with a wavelength of 1030 nm, a pulse duration of 270 fs, a repetition rate of 100 kHz, and a pulse energy of 30 μJ was used for ablation. To ensure uniform target processing, the laser beam was scanned along a spiral trajectory at a speed of 4 m/s using a galvanometric scanner.

The resulting colloid was centrifuged at 15,000 g for 15 min. Large particles (larger than 10 nm) pelleted during this step were discarded. Only the supernatant, containing the smaller AuNP fraction, was used for subsequent steps.

Next, an iron target was ablated in an ablation cell containing 25 mL of the AuNP colloid (0.32 mg/mL). This produced iron oxide nanoparticles that served as cores in the Fe_3_O_4_@Au nanostructures. The ablation of the iron target was performed using the same laser but with a reduced pulse energy of 5 μJ; the synthesis lasted 13 min.

Finally, magnetic separation was performed to isolate the Fe_3_O_4_@Au nanoparticles. A neodymium magnet was placed against the side wall of the vessel containing the colloidal solution, causing magnetic nanoparticles to sediment near the magnet. After 10 min, the liquid was carefully removed, leaving 300–500 μL near the magnet. The nanoparticles retained in this volume were redispersed in 5 mL of liquid for further use.

### 4.2. Functionalization of Fe_3_O_4_@AuNP Surface

The surface of the Fe_3_O_4_@Au nanoparticles was functionalized using 15 kDa polyethylene glycol (PEG). The polymer was conjugated to the gold satellites via a stable Au–S bond, employing lipoic acid (LA) as a linker.

The LA–PEG conjugate was prepared in advance. First, 350 µM lipoic acid, 350 µM 1-ethyl-3-(3-dimethylaminopropyl)carbodiimide (EDC), and 17.5 µM 4-dimethylaminopyridine (DMAP) were dissolved in dichloromethane (DCM). The solution was degassed under argon for 1 h at 30 °C in a water bath. The mixture was then cooled to 0 °C in an ice bath, and 330 µM PEG pre-dissolved in 5 mL of DCM was added. The reaction was stirred at 0 °C for 1 h and then left at room temperature for 20 h.

After the reaction, the mixture was extracted with an equal volume of water, vortexed, and centrifuged at 4225 g for 1 min. The aqueous phase was discarded, and the extraction was repeated 4–5 times until a completely transparent organic phase was obtained. Residual water was removed using a rotary evaporator at 45 °C for 40 min. The mixture was then allowed to cool for 15 min, yielding yellowish crystals. These crystals were dissolved in 4 mL of DCM. Finally, the product was reprecipitated by adding the DCM solution to 50 mL of diethyl ether, followed by centrifugation. The ether supernatant was discarded, and the precipitate was dried overnight. The resulting LA–PEG conjugate was used for nanoparticle coating.

Before coating, the stock colloidal solution of the Fe_3_O_4_@AuNPs was placed in an ultrasonic bath for 10 min. An aliquot was then taken to determine the gold concentration using ICP-OES. Based on the measured gold concentration, the total gold surface area in the colloid was calculated, and the required amount of LA–PEG was prepared, assuming one molecule per 1 nm^2^ of gold surface. The LA–PEG was dissolved in deionized water and added dropwise to the Fe_3_O_4_@AuNP solution under continuous sonication. The mixture was then left for 24 h at room temperature under gentle stirring.

After incubation, the colloidal solution was centrifuged in 5 mL aliquots (Falcon tubes) at 4225 g for 20 min. The supernatant was discarded, leaving 50 µL of the pellets at the bottom of each tube. The pellets were combined and transferred into a dialysis bag with a 20 kDa molecular weight cutoff for 2 days. Following dialysis, the solution was filtered through a 0.22 µm pore-size filter to obtain a sterile aqueous colloid of laser-ablated Fe_3_O_4_@Au core–satellite nanoparticles.

### 4.3. Characterization of Fe_3_O_4_@AuNPs

The characterization of the final Fe_3_O_4_@AuNP colloid was performed using several complementary techniques.

Scanning electron microscopy (SEM) was performed to examine the morphology and structure of the synthesized nanoparticles using an MAIA 3 scanning electron microscope (Tescan, Brno, Czech Republic). Samples for electron microscopy were prepared by applying 2 μL of the nanoparticle solution to a cleaned silicon substrate, followed by drying under normal conditions.

UV–Vis spectrophotometry was carried out to record the absorption spectrum. For the analysis, an aliquot of the colloidal solution was diluted with distilled water to an optical density between 0.2 and 1.4. The spectrum was recorded in the range of 200–800 nm using a Cary 50 spectrophotometer (Varian, Palo Alto, CA, USA) in a quartz cuvette with an optical path length of 10 mm at 20 °C.

Energy-dispersive X-ray spectroscopy (EDX) was performed to confirm the elemental composition of the nanoparticles with an X-Act EDX module (Oxford Instruments, Abingdon, UK) at an accelerating voltage of 20 keV. Samples were prepared by placing 2 µL of the colloidal solution onto a silicon substrate and drying under ambient conditions.

Fourier-transform infrared (FTIR) spectra were recorded using a laboratory IR Fourier spectrometer FSM-2203 (Infraspek, St. Petersburg, Russia) equipped with an ATR (Attenuated Total Reflection) attachment; the spectra were collected in the range 650–4000 cm^−1^ with 32 scans. Before analysis, the sample was dried to a powder state.

Dynamic light scattering (DLS) and zeta potential were applied to determine the hydrodynamic diameter and surface charge. The measurements were conducted using a Linkoptik Nanolink SZ instrument (Linkoptik, Zhuhai, China). Zeta potential was assessed in phosphate-buffered saline (PBS, pH 7.4) after 15 min of incubation. Hydrodynamic size and zeta potential were measured in three series, each comprising 15–20 individual measurements.

Inductively coupled plasma optical emission spectrometry (ICP-OES) was employed to measure the concentrations of gold and iron using a high-resolution PlasmaQuant 9100 Series spectrometer (Analytik Jena, Jena, Germany) and standard calibration solutions. For sample preparation, colloidal aliquots were digested in a mixture of nitric and hydrochloric acid (aqua regia) using an Ethos Easy microwave decomposition system (MILESTONE, Sorisole, Italy) at 190 °C and 15 bar.

MRI relaxivity was measured with a Nanoscan 3T MRI scanner (Mediso, Budapest, Hungary) at 20 °C. For r_1_ relaxivity, a Multi Inversion Recovery Fast Spin Echo (Multi-IR FSE) sequence was applied in the coronal orientation with the following parameters: slice thickness, 1.5 mm; interslice gap, 0.2 mm; field of view (FOV), 63 × 63 mm; matrix, 192 × 192; in-plane resolution, 0.34 × 0.34 mm; TR = 7700 ms; TE = 11 ms; TI values of 50, 100, 200, 300, 400, 500, 600, 700, 800, 900, 1000, 1200, 1400, 1600, 1800, 2000, and 2500 ms; and NEX = 1.

For r_2_ relaxivity, a Multi Echo Spin Echo (Multi-Echo SE) sequence was used in the coronal orientation with the same geometric parameters, and the following sequence parameters: TR = 4600 ms; TE = 15, 30, 45 … 480 ms; and NEX = 1. Image processing and calculation of r_1_ and r_2_ relaxivity values were performed using InterView Fusion software version 3.11.000.9005 (Mediso, Budapest, Hungary).

### 4.4. CT Radiodensity Calibration

To establish a calibration curve for quantifying gold concentration from CT images, a phantom study was performed according to previously reported protocols [55,56]. The phantom consisted of tubes containing colloidal Fe_3_O_4_@AuNP solutions at known gold concentrations (1–34 mg/mL), along with a water-filled tube as a reference. The actual gold concentrations were confirmed by ICP-OES using a high-resolution PlasmaQuant 9100 Series spectrometer (Analytik Jena, Jena, Germany). Before ICP-OES analysis, samples were digested in aqua regia (a 1:3 (*v*/*v*) mixture of concentrated nitric and hydrochloric acids).

The phantom was scanned using the CT modality of a VECTor6 preclinical trimodal scanner (MiLabs, Utrecht, The Netherlands). Radiodensity (Hounsfield units, HU) within each tube was quantified using PMOD software (PMOD Technologies LLC, Zurich, Switzerland), and the HU value of pure water was subtracted. The resulting increase in radiodensity (ΔHU) was plotted against the gold concentration ([Au], mg/mL). The relationship was fitted with a linear function.

### 4.5. Biodistribution Study by CT and MRI

All procedures involving animals were performed in accordance with local ethical regulations and approved by the institutional ethical committee (protocol No. 2025-1i, issued on 21 January 2025).

For the biodistribution study, healthy female C57BL/6 mice were divided into an experimental group (n = 6) and a control group (n = 8). Animals in the experimental group first underwent CT and MRI to acquire baseline (native) images and then received an intravenous injection of Fe_3_O_4_@AuNPs at a dose of 730 ± 40 mg Au/kg, corresponding to 82 ± 5 mg Fe/kg. The control group received an intravenous injection of saline.

The first post-injection CT and MRI scans were performed as soon as technically possible after Fe_3_O_4_@AuNP administration, i.e., CT approximately 5 min post-injection, followed by MRI approximately 20 min post-injection. Thereafter, all animals in the experimental group were examined by CT, followed immediately by MRI, at the following time points: 1 h, 3 h, 24 h, 3 days, 7 days, 14 days, 1 month, and then once a month for one year.

During scanning (in both modalities), animals were maintained under anesthesia induced by a 2% isoflurane–air mixture.

CT was performed using the CT modality of a VECTor6 preclinical trimodal scanner (MiLabs, Utrecht, The Netherlands). The following parameters were used: X-ray tube voltage of 55 kV, tube current of 0.21 mA, exposure time per projection of 75 ms, and a rotation step of 0.5°, with one projection acquired per step. Images were reconstructed with an isotropic voxel size of 0.1 mm. Image reconstruction was performed using MiLabs Rec 12.00 software (MiLabs, Utrecht, The Netherlands).

CT images were used for quantitative assessment of gold concentration in organs and tissues. Volumes of interest (VOIs) were manually delineated in key organs, including the heart, liver, spleen, kidneys, brain, and muscle. Gold content within each organ was quantified using the calibration curve described in Section 4.4 and expressed in mg/cm^3^. Post-processing and image analysis were performed using PMOD 4.205 software (PMOD Technologies LLC, Zurich, Switzerland). CT images in the figures are displayed with a window range of –500 to +600 HU.

MRI was performed using a NanoScan 3T scanner (Mediso, Budapest, Hungary) in the coronal plane with a Fast Spin-Echo sequence. The following parameters were used: TR = 555 ms, TE = 13 ms, echo train length (ETL) = 4, field of view (FOV) = 100 × 32 mm, in-plane resolution = 0.2 × 0.2 mm, slice thickness = 0.8 mm, and interslice gap = 0.1 mm. The scan covered the entire body of the mouse.

MRI images were evaluated qualitatively using InterView Fusion software version 3.11.000.9005 (Mediso, Budapest, Hungary).

### 4.6. ICP-OES

At the experimental endpoint, all mice (experimental and control) were euthanized after the final 365-day scan. Organs (spleen, liver, kidneys, lung, skin, muscle, brain) were collected for gold and iron concentration analysis. From these same animals, tissue samples were first taken for histological examination, and the remaining portions of the organs were processed for ICP-OES.

For elemental analysis, samples were mineralized by microwave-assisted digestion in an Ethos Easy system (MILESTONE, Sorisole, Italy) using a mixture of 1 mL concentrated HNO_3_, 3 mL concentrated HCl, and 1 mL H_2_O_2_. The temperature was gradually increased to 190 °C over 30 min, followed by a 10 min incubation at 190 °C. After cooling to 50 °C, the digestate was transferred to 50 mL Falcon tubes, supplemented with 50 µL of Triton X-100, and diluted to 10 mL with deionized water.

Gold and iron concentrations were determined by inductively coupled plasma optical emission spectrometry (ICP-OES) using a PlasmaQuant 9100 Series spectrometer (Analytik Jena, Germany). Measurements were performed in triplicate at two emission wavelengths for each element: 242.795 nm and 267.595 nm for gold; 259.940 nm and 238.204 nm for iron. A calibration curve was established using standard solutions of both elements, yielding a linear correlation (R^2^ > 0.99). Final concentrations were calculated based on the dilution factors applied during sample preparation. Data are presented as mean ± SD. Statistical comparisons between the control and Fe_3_O_4_@AuNP-treated groups were performed using Welch’s *t*-test. A *p*-value < 0.05 was considered statistically significant.

### 4.7. Toxicity Evaluation

Toxicity was evaluated in the same mice used for the biodistribution study. Two groups of female C57BL/6 mice were used: an experimental group (n = 6) that received a single intravenous injection of Fe_3_O_4_@AuNPs (730 ± 40 mg Au/kg, 82 ± 5 mg Fe/kg) and a control group (n = 8) that received an intravenous injection of saline. Both groups were monitored for signs of distress immediately after injection and underwent clinical examination and body weight measurements three times per week for the first three weeks and then once every two weeks for the remaining one-year observation period. Body weight data are presented as mean ± SD.

At the end of the study, after the final CT scan, all mice were euthanized by isoflurane overdose. Blood was collected for biochemical analysis, including measurements of creatinine, urea, alanine aminotransferase (ALT), aspartate aminotransferase (AST), total and direct bilirubin, and amylase. The analysis was performed using a Q-VET automatic veterinary biochemical analyzer (GOLDSITE, Shenzhen, China). Data are presented as mean ± SD. Statistical comparisons between the control and Fe_3_O_4_@AuNP-treated groups were performed using Welch’s *t*-test. A *p*-value < 0.05 was considered statistically significant.

Immediately after blood collection, tissue samples from the liver, spleen, kidneys, lungs, and skin were harvested and fixed in 10% neutral buffered formalin for histological processing. Samples were fixed at room temperature for 24 h at a volume ratio of 10:1 (formalin to tissue). Subsequently, they were processed in an STP-120 histoprocessor (Epredia, Portsmouth, NH, USA), embedded in paraffin blocks using a TES99 system (Medite, Burgdorf, Germany), and sectioned into 2–5 μm slices with a microtome CUT6062 (Slee, Mainz, Germany). Sections were mounted on glass slides, dried, and stained with hematoxylin and eosin using a Gemini AS automated stainer (Thermo Fisher Scientific, Waltham, MA, USA). Finally, the slides were coverslipped and examined via light microscopy using a BX46 microscope (Olympus, Hachioji, Japan).

## 5. Conclusions

This study provides a long-term (one-year) evaluation of the biodistribution and safety profile of core–satellite Fe_3_O_4_@AuNPs following high-dose administration to mice. CT, MRI, and ICP-OES confirmed that after intravenous injection, the nanoparticles accumulated predominantly in the liver and spleen. Subsequently, MRI revealed changes consistent with a slow loss of magnetic Fe_3_O_4_ material, and CT showed a moderate decrease in gold content in the liver. Biochemical and histological analyses performed one year post-administration detected no evidence of organ damage, except for mild age-related alterations. Future research directions may include assessment of transient toxicity (within 1–14 days) and further studies in tumor-bearing mice to evaluate the potential for oncological applications.

## Figures and Tables

**Table 1:** Gold and iron concentrations in organs of control (saline-treated) and Fe_3_O_4_@AuNP-treated mice one year after intravenous administration.

Organ	Fe_3_O_4_@AuNPs			Saline		*p*-Value [Fe]
	CT	ICP-OES		ICP-OES		
	[Au], μg/cm^3^	[Au], μg/g	[Fe], μg/g	[Au], μg/g	[Fe], μg/g	
Spleen	7337 ± 1396	9660 ± 1840	1551 ± 183	Not detected	1142 ± 267	0.005 **
Liver	3856 ± 536	3129 ± 265	515 ± 26	Not detected	205 ± 10	<0.001 ***
Kidney	Not detected	143 ± 10	102 ± 8	Not detected	79 ± 5	<0.001 ***
Lung	Not assessed	115 ± 23	94 ± 9	Not detected	80 ± 3	0.009 **
Skin	Not assessed	95 ± 40	21 ± 2	Not detected	28 ± 5	0.005 **
Muscle	Not detected	53 ± 18	18 ± 3	Not detected	20 ± 2	0.255
Brain	Not detected	3 ± 4	29 ± 2	Not detected	24 ± 1	0.002 **

Data are presented as mean ± SD. Statistical significance was determined using Welch’s *t*-test. ** *p* < 0.01, *** *p* < 0.001 vs. control group.

**Table 2:** Serum biochemical parameters in control (saline-treated) and Fe_3_O_4_@AuNP-treated mice one year after intravenous administration. Data are presented as mean ± SD.

Parameter	Fe_3_O_4_@AuNPs	Saline	*p*-Value
Creatinine (µmol/L)	91.1 ± 4.1	85.8 ± 4.2	0.035 *
Urea (mmol/L)	10.2 ± 1.5	10.8 ± 1.7	0.512
ALT (U/L)	76.5 ± 9.9	84.7 ± 5.3	0.105
AST (U/L)	152.1 ± 28.3	125.8 ± 15.1	0.076
Total bilirubin (µmol/L)	3.7 ± 0.2	3.9 ± 0.4	0.322
Direct bilirubin (µmol/L)	1.2 ± 0.2	1.4 ± 0.2	0.993

* *p* < 0.05.

## Data Availability

Dataset available on request from the authors.

## Supplementary Materials

The following Supporting Information can be downloaded at: https://www.mdpi.com/article/10.3390/ijms27146147/s1.

## Abbreviations

The following abbreviations are used in this manuscript:

Fe_3_O_4_@AuNPs	iron oxide–gold nanoparticles
AuNPs	gold nanoparticles
MRI	magnetic resonance imaging
CT	computed tomography
HU	Hounsfield units
RES	reticuloendothelial system
PEG	polyethylene glycol
LA	lipoic acid
SEM	scanning electron microscopy
EDX	energy-dispersive X-ray spectroscopy
FTIR	Fourier-transform infrared spectroscopy
ICP-OES	inductively coupled plasma optical emission spectrometry
DLS	dynamic light scattering
PBS	phosphate-buffered saline
ALT	alanine aminotransferase
AST	aspartate aminotransferase
ROS	reactive oxygen species
