# Supplementary material for: Long-Term Biodistribution of Fe3O4@Au Core–Satellite Nanoparticles Assessed by CT and MRI In Vivo
Source: Int J Mol Sci. 2026 Jul 9;27(14):6147. doi: 10.3390/ijms27146147 (PMC13411982; doi:10.3390/ijms27146147)
Supplement: Supplementary file 1 [file ijms-27-06147-s001.zip › ijms-4370314-supplementary.pdf]

Long-Term Biodistribution of Fe_3O_4@Au Core–Satellite Nanoparticles Assessed by CT and MRI In Vivo

Kristina Shpakova [1,2], Vsevolod Skribitsky [1,2,3], Yulia Finogenova [1,2*], Anton Kasianov [1,3], Alexey Lipengolts [1,2,3*], Angelina Skribitskaya [3], Anna Smirnova [1,4], Artem Laktionov [3], Sergey Klimentov [3] and Elena Grigorieva [1,2]

[1] N.N. Blokhin National Medical Research Center of Oncology, Moscow 115522, Russia; skvseva@yandex.ru (V.S.); a_kasianov@mail.ru (A.K.); shpakova.k.e@gmail.com (K.S.); smirn-ova@mail.ru (A.S.); b-f.finogenova@yandex.ru (Y.F.); lipengolts@mail.ru (A.L.)
[2] Institute for Physics and Engineering in Biomedicine, Russian Academy of Sciences, Moscow 119991, Russia
[3] Engineer and Physics Institute of Biomedicine, National Research Nuclear University MEPhI, Moscow 115409, Russia; sav1998@list.ru (A.S.); aalaktionov@mephi.ru (A.L.)
[4] The Loginov Moscow Clinical Scientific Center, Moscow 111123, Russia
* Correspondence: ju.finogenova@ronc.ru (Y.F.); lipengolts@ronc.ru (A.L.)

Supplementary materials

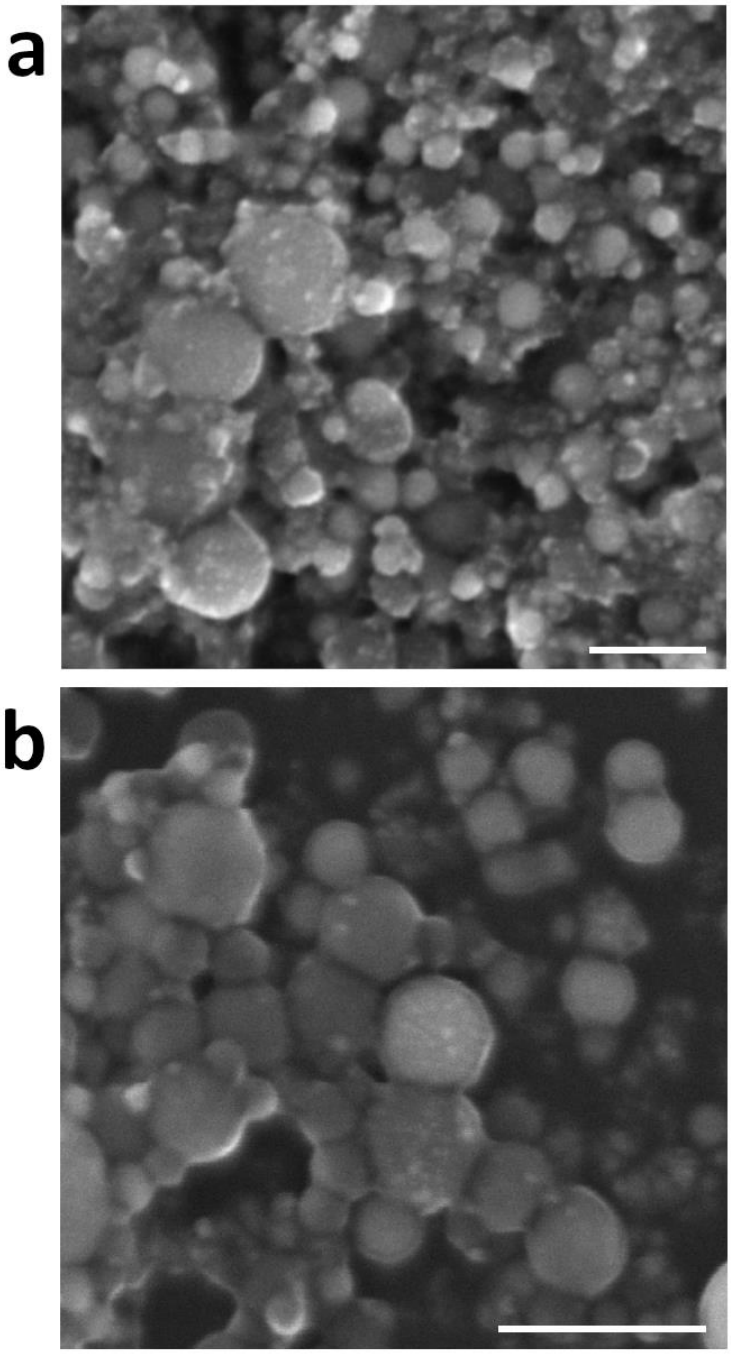

Figure S1. Representative SEM images of Fe_3O_4@AuNPs before and after PEG functionalization: (**a**) As-prepared bare nanoparticles; (**b**) Nanoparticles after PEG coating. Scale bar = 200 nm on both images.

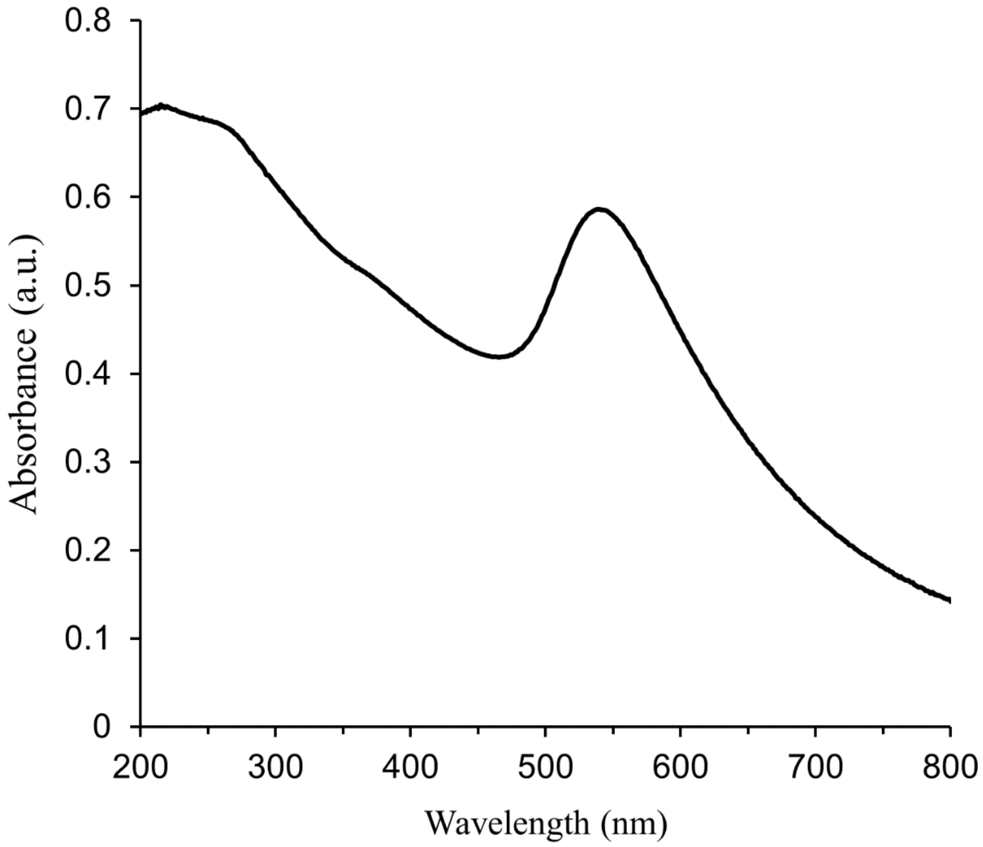

Figure S2. UV-Vis absorption spectrum of Fe_3O_4@AuNPs. The spectrum exhibits a surface plasmon resonance peak at 540 nm, originating from the gold satellites decorating the iron oxide core.

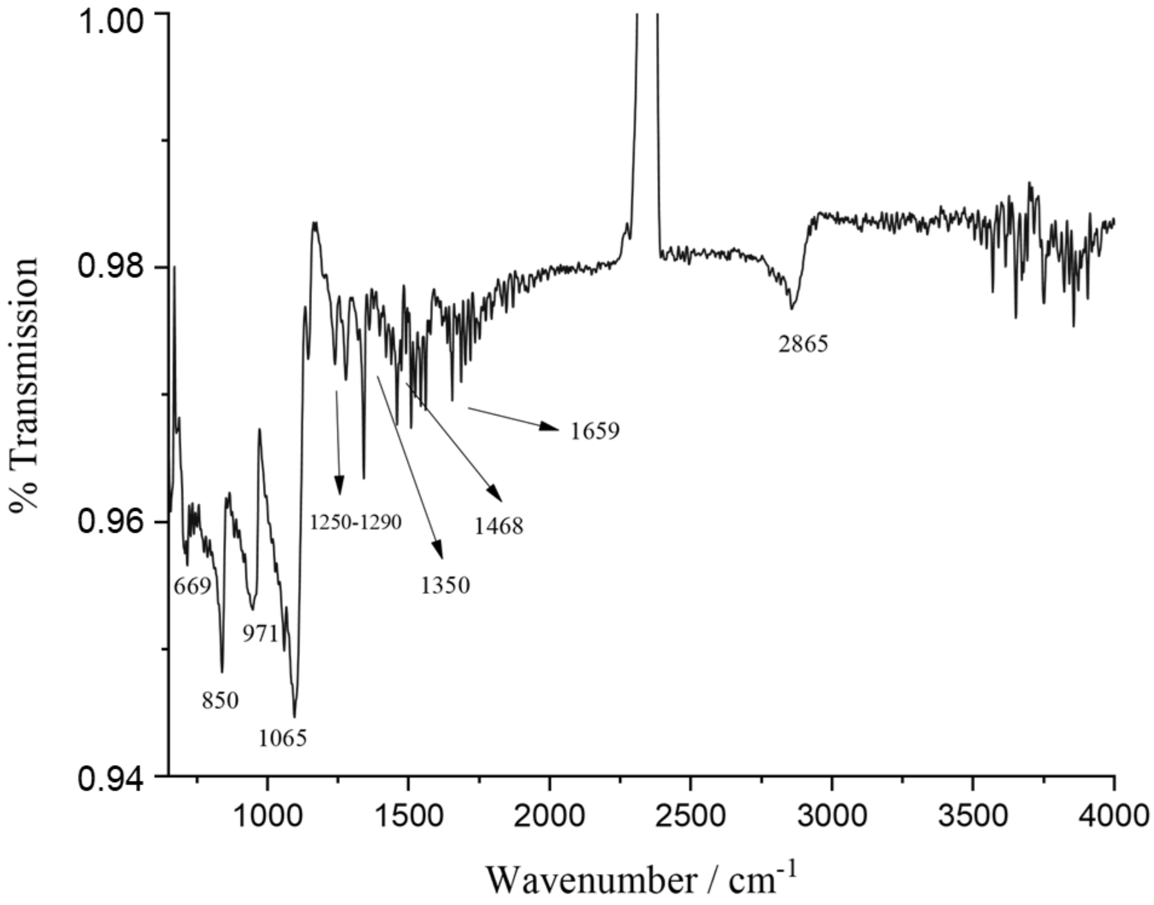

Figure S3. FTIR spectrum of PEG-LA coated Fe_3O_4@AuNPs. Characteristic bands of polyethylene glycol (C–O–C at ~971 and 1065 cm^{-1}, CH_2 at ~1350 and 1468 cm^{-1}), an amide I band at ~1659 cm^{-1} confirming the amide linkage, and a C–S band at ~669 cm^{-1} indicating thiolate binding to gold.

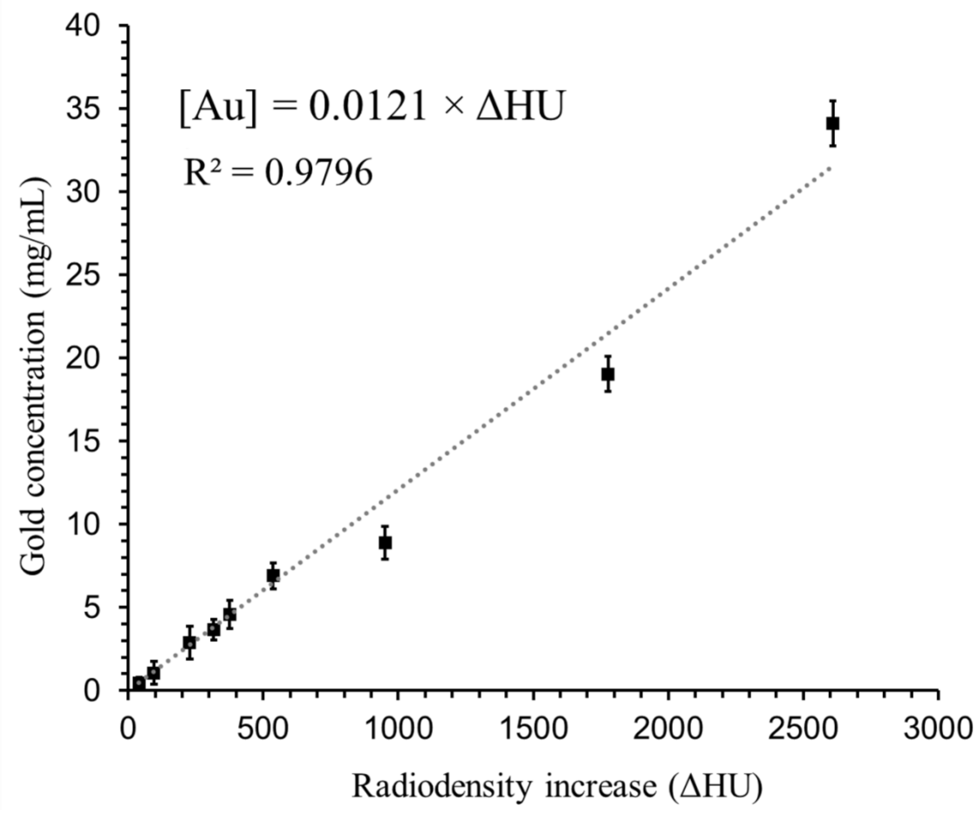

Figure S4. Calibration plot relating the increase in radiodensity (ΔHU) to gold concentration ([Au], mg/mL).

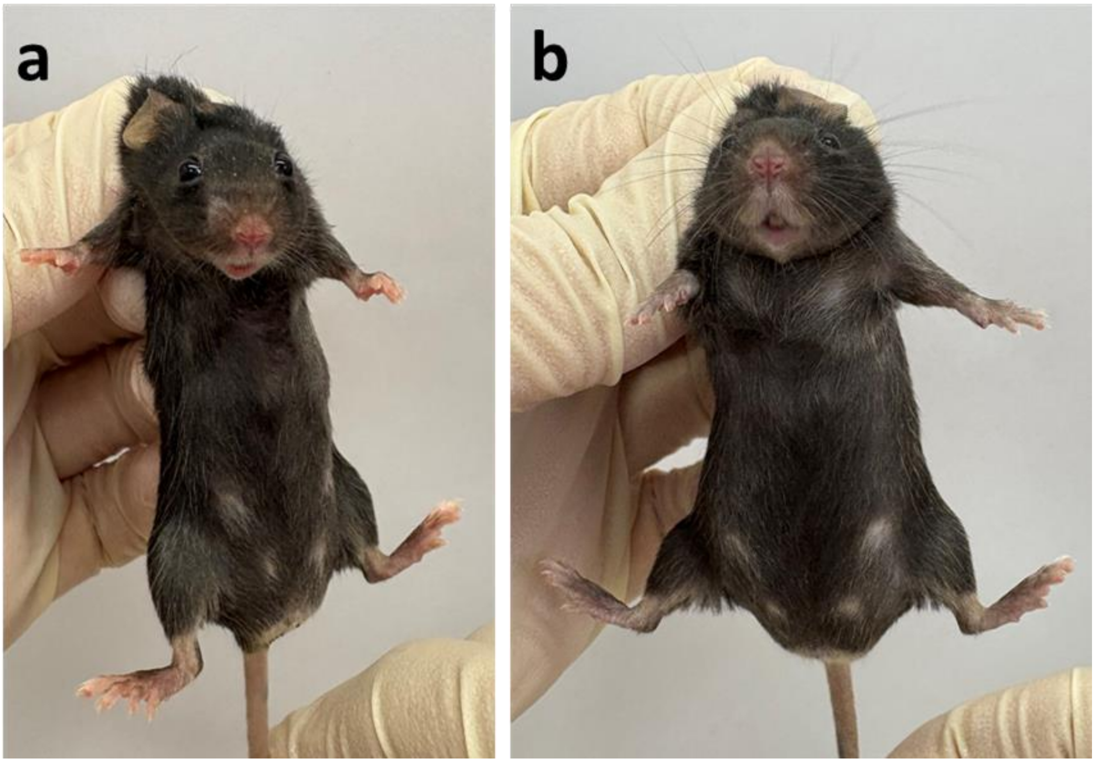

Figure S5. Skin discoloration in Fe_3O_4@AuNP-treated mouse: (**a**) Control mouse (saline injection); (**b**) Fe_3O_4@AuNP-treated mouse showing slight darkening of the paws and tail.

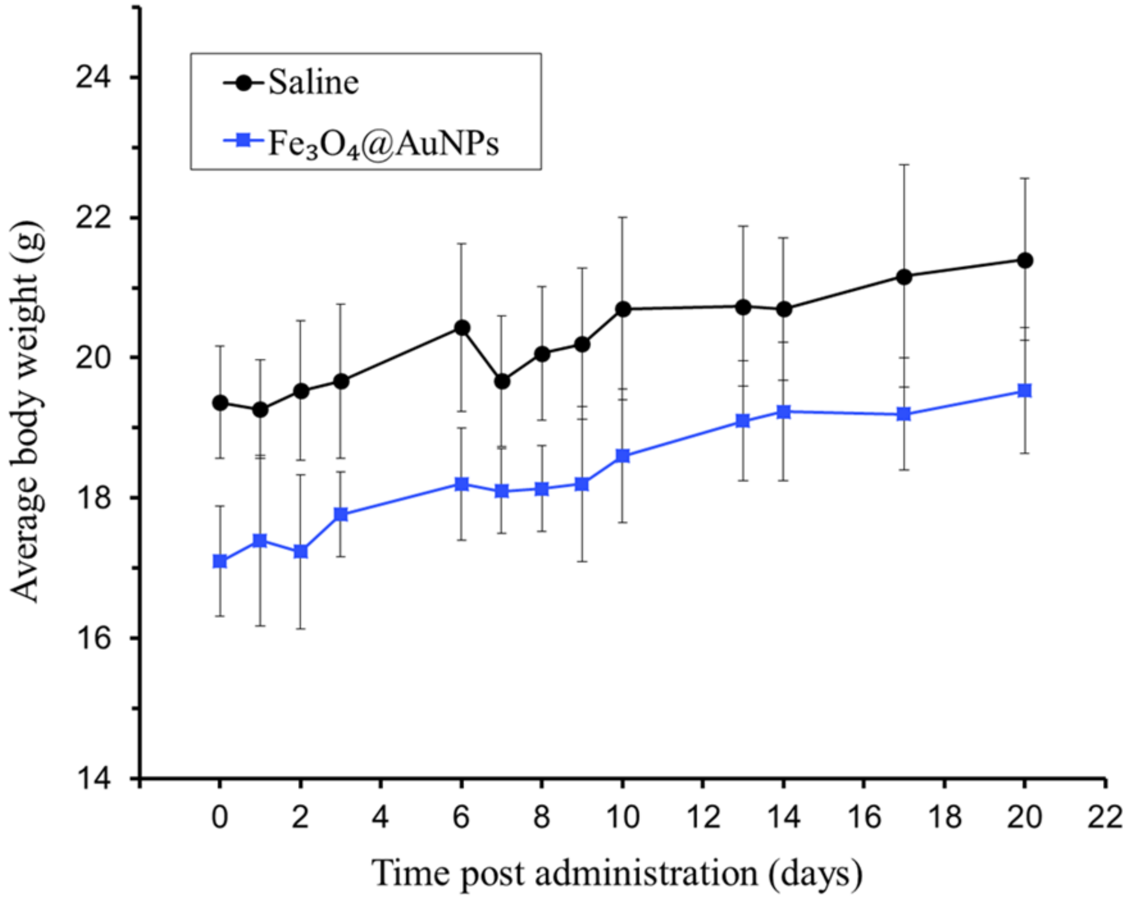

Figure S6. Short-term body weight dynamics (first 21 days). Absolute body weight of control (saline-injected) and Fe_3O_4@AuNP-treated mice over the first three weeks after intravenous injection. Data are presented as mean ± SD.

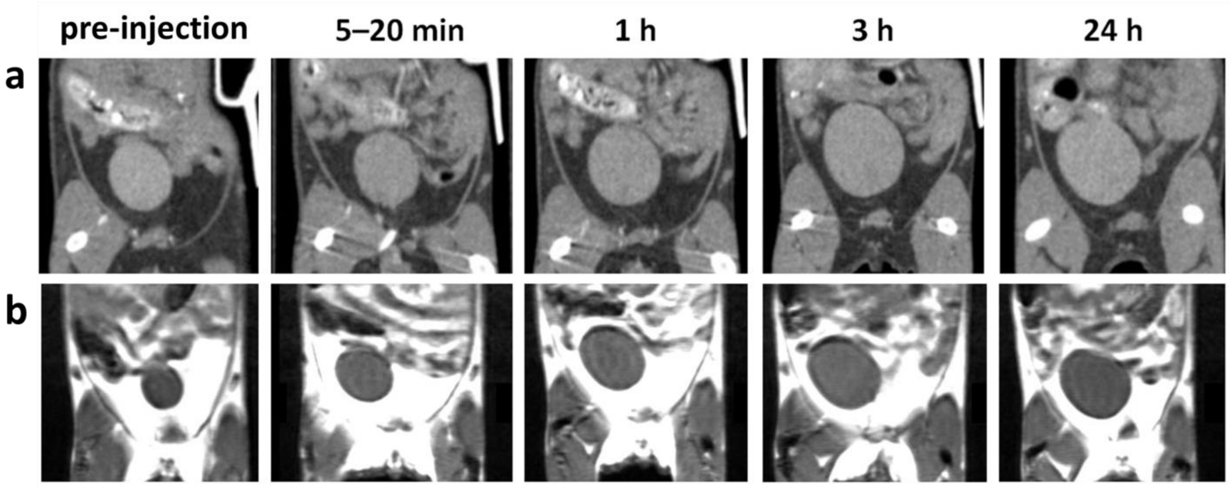

Figure S7. Coronal CT and MRI images of the urinary bladder: (**a**) CT images before injection (native) and at several time points after Fe_3O_4@AuNP injection up to 24 h; (**b**) MRI images at the same time points. No contrast enhancement is visible in either modality, indicating absence of renal excretion.

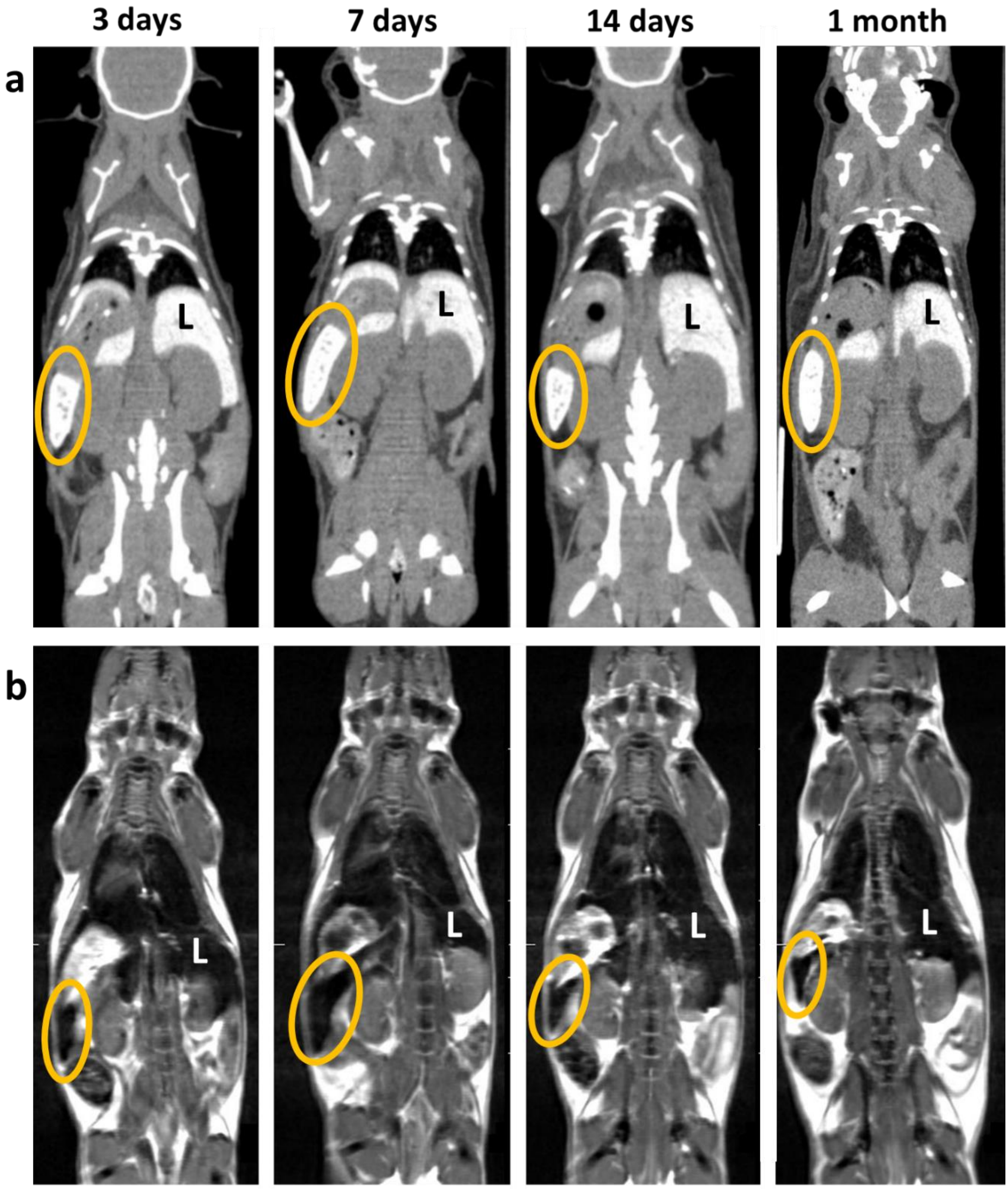

Figure S8. CT and MRI images of mouse organs (coronal views): (**a**) CT images of the liver, spleen, and kidneys from 3 days to 1 month after Fe_3O_4@AuNP injection; (**b**) MRI images of the same organs at the same time points. Orange ring indicates the spleen, the letter L indicates the liver.

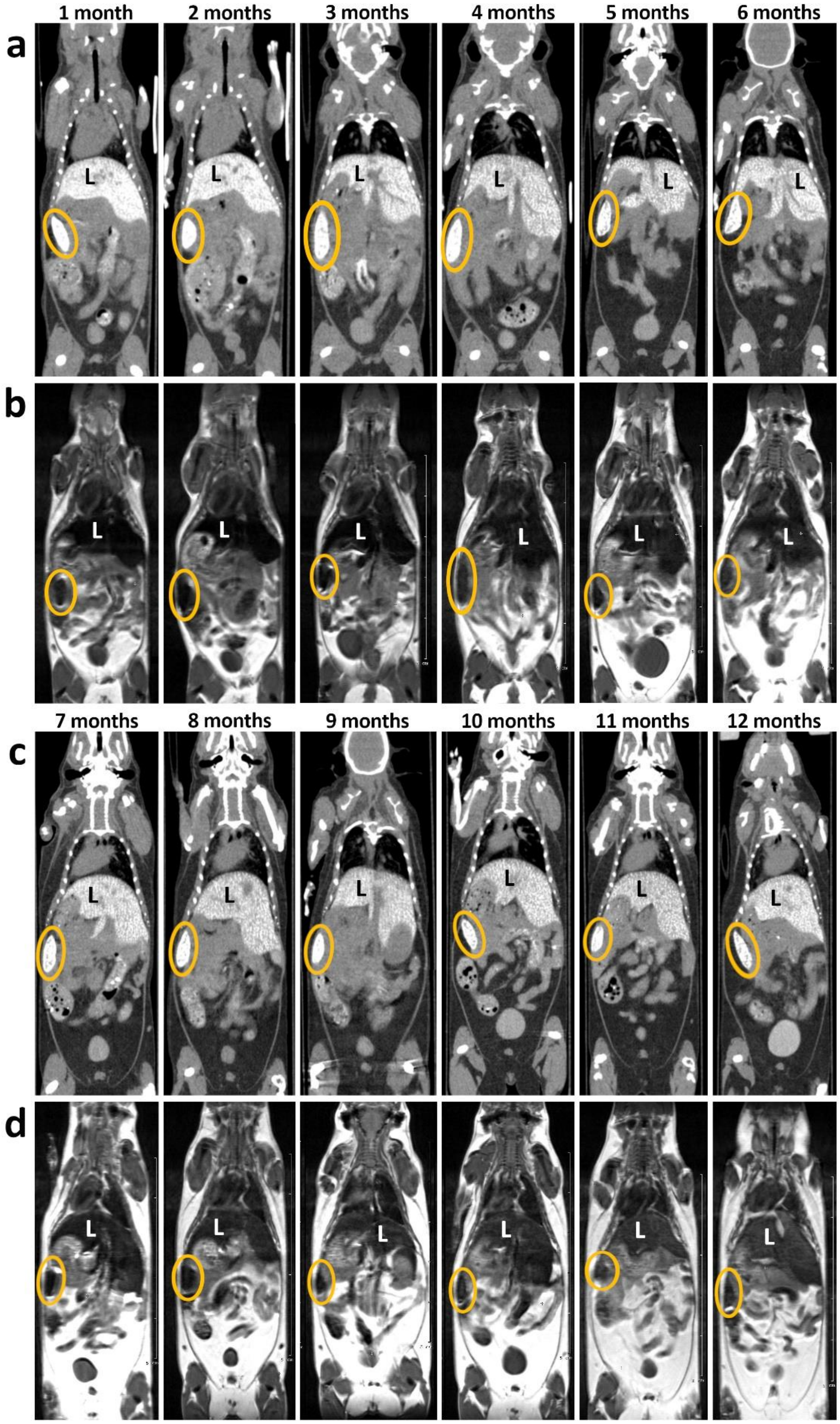

Figure S9. CT and MRI images of mouse liver and spleen (coronal views): (**a, c**) CT images from 1 to 12 months after Fe_3O_4@AuNP injection (all monthly time points); (**b, d**) MRI images of the same organs at the same time points. Orange ring indicates the spleen, the letter L indicates the liver.

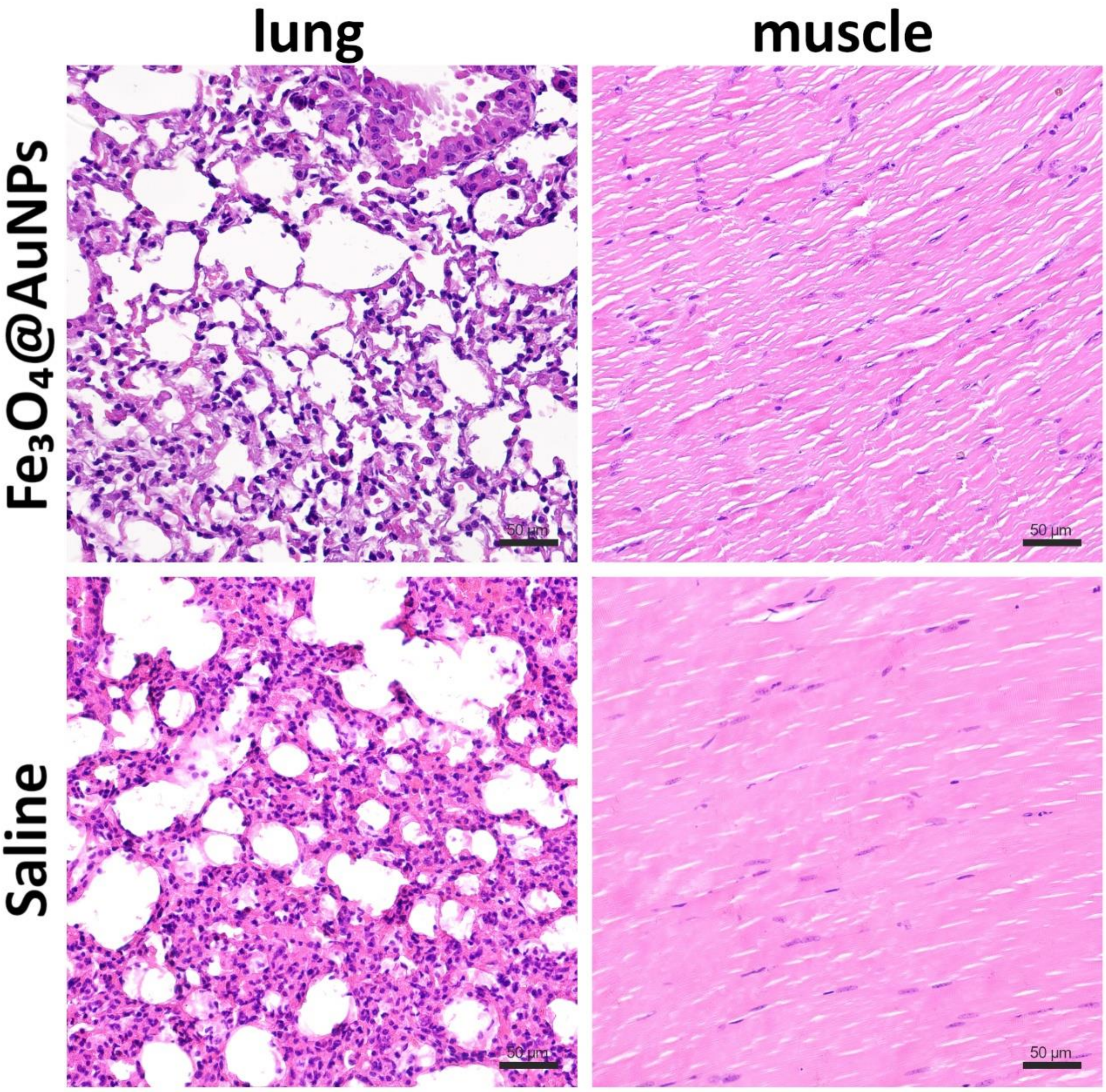

Figure S10. Representative histological sections of the lung and muscle from Fe_3O_4@AuNP-treated and control mice. H&E staining. Scale bar = 50 μm.
